# Myocardial Bridging Leading to Cardiac Collapse in a Marathon Runner

**DOI:** 10.3390/jcdd9070200

**Published:** 2022-06-24

**Authors:** André Alexandre, Pinheiro Vieira, André Dias-Frias, Anaisa Pereira, Andreia Campinas, David Sá-Couto, Bruno Brochado, Isabel Sá, João Silveira, Severo Torres

**Affiliations:** 1 Centro Hospitalar Universitário do Porto, 4099-001 Porto, Portugal; pinheirovieira@sapo.pt (P.V.); andre.frias1992@gmail.com (A.D.-F.); anaisaalexandra@icloud.com (A.P.); andreia.campinas23@hotmail.com (A.C.); david.sa.couto@gmail.com (D.S.-C.); bruno_brochado@hotmail.com (B.B.); isabelpsa@gmail.com (I.S.); jbrumsilveira@gmail.com (J.S.); severotorres.cardiologia@chporto.min-saude.pt (S.T.); 2 ICBAS–School of Medicine and Biomedical Sciences, Porto University, 4050-313 Porto, Portugal

**Keywords:** myocardial bridging, coronary vessel anomaly, cardiac collapse, exertional syncope, marathon running, arrhythmia, coronary computed tomography angiography, strain imaging

## Abstract

Myocardial bridging (MB) is a congenital coronary anomaly, which is defined as cardiac muscle overlying a portion of a coronary artery. Although traditionally considered benign in nature, increasing attention is being given to specific subsets of MB. Sports medicine recognizes MB as a cause of sudden death among young athletes. We present a case of a 30-year-old man who suddenly collapsed during a marathon running. Diagnostic workup with coronary computed tomography angiography revealed the presence of three simultaneous myocardial bridges in this patient, possibly explaining the exercise-induced syncope. The other diagnostic tests excluded seizures, cranioencephalic lesions, ionic or metabolic disturbances, acute coronary syndromes, cardiomyopathies, myocarditis, or conduction disturbances. Exertional syncope is a high-risk complaint in the marathon runner. In the context of intense physical activity, the increased sympathetic tone leading to tachycardia and increased myocardial contractility facilitates MB ischemia. In this illustrative case, the patient’s syncope might probably be associated with an ischemia-induced arrhythmia secondary to MB and potentiated by dehydration in the context of prolonged stress (marathon running). In conclusion, this case highlights that MB may be associated with dangerous complications (myocardial ischemia and life-threatening ventricular arrhythmias), particularly during intense physical activity and in the presence of a long myocardial bridge.

## 1. Introduction

Myocardial bridging (MB) is a congenital coronary anomaly, which is defined as cardiac muscle overlying a portion of a coronary artery [1,2]. The true prevalence of MB is not accurately known; however, MB is likely present to some degree in approximately one in three adults [1]. Although traditionally considered benign in nature, increasing attention is being given to specific subsets of MB [1]. Sports medicine recognizes MB as a cause of sudden death among young athletes, but its clinical relevance is still under debate [2,3,4,5]. We present a case of a 30-year-old man who suddenly collapsed during a marathon run. Diagnostic workup with coronary computed tomography angiography revealed the presence of three simultaneous myocardial bridges in this patient, possibly explaining the exertional syncope.

## 2. Case Report

### 2.1. History of Presentation

A 30-year-old man suddenly collapsed during a marathon run (at approximately 17 miles). The syncopal event was witnessed, and cardiopulmonary resuscitation (CPR) maneuvers were initiated by laypeople. The rhythm of cardiac arrest was not documented due to the inexistence of an automated external defibrillator. After approximately 10 min of CPR and upon arrival of emergency medical services, the patient was found to have returned to spontaneous circulation. Since he had a Glasgow Coma Scale of 4, he was intubated and then admitted to the emergency department. On admission, generalized tonic-clonic seizures were documented, resolving with short-acting benzodiazepine.

### 2.2. Past Medical History

The patient had a history of palpitations diagnosed as anxiety disorder since childhood and recurrent dislocation of the left shoulder. He had no history of any other significant medical illness or previous chest pain. He was not a smoker and did not report the use of drugs. No sudden cardiac death was reported in his family. The patient started running at the age of 25. He had a training routine of 5 days/week, 6–9 miles per workout, at a pace of 8 min/mile. At age 27, he ran his first half marathon (13 miles) and decided to prepare himself for a marathon run. Subsequently, he began to run longer distances. The month before running the marathon, he ran the longest distance of 19 miles. In one of his running workouts, he had an episode of dizziness and lightheadedness that led him to stop running at 12 miles (without total loss of consciousness).

### 2.3. Differential Diagnosis

The differential diagnosis for this clinical presentation included acute coronary syndromes, malignant arrhythmias, myocarditis, acute cerebrovascular accidents, seizure disorders, and ionic or metabolic disturbances.

### 2.4. Investigations

**Figure 1:**
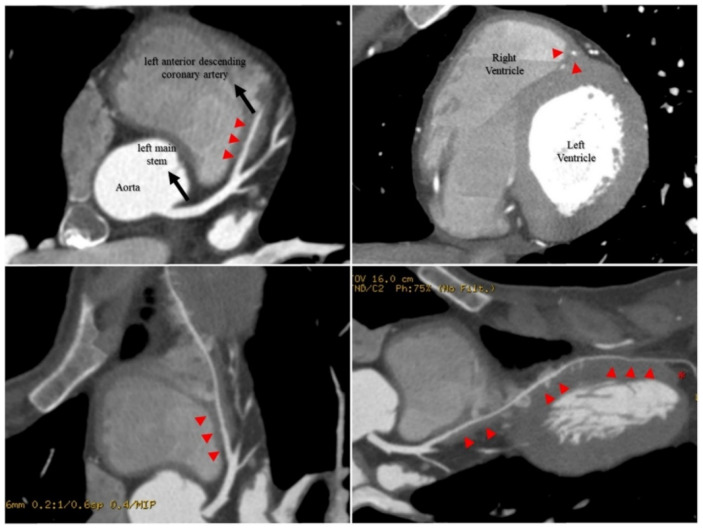
LAD intramyocardial segment shown in CCTA imaging. CCTA revealed an extensive LAD intramyocardial segment (red arrowheads) starting at its proximal segment and running adjacent to the right ventricular free wall in the mid-distal portion, normalizing its usual anatomical position at the apex level (red asterisk). CCTA: coronary computed tomography angiography; LAD: left anterior descending coronary artery.

On hospital admission, considering the presence of generalized tonic-clonic seizures, urgent cranial and cervical CT scans, CT angiography of the cerebral arteries, skeletal and spine radiographs, and brain MRI were performed, ruling out acute structural lesions, recent fractures, or spinal injuries. No ionic or metabolic disturbances were documented. ECG showed sinus rhythm (69 beats/min) with no ST-T changes. He had no significant increase in cardiac biomarkers (maximum levels of high-sensitivity troponin T, creatine kinase, and myoglobin were 0.115 ng/mL [reference values 0.000–0.014], 328 U/L (24–204), and 212 μg/L (28–72), respectively). Toxic substances were excluded. Echocardiographic evaluation was unremarkable, with normal systolic and diastolic function, normal-sized cardiac cavities, no valvular morphologic changes, and no pericardial effusion. After exclusion of potential life-threatening diagnosis, the patient was hospitalized for further diagnostic workup. He was extubated the day after admission. During hospitalization, continuous in-hospital ECG monitoring showed no rhythm disturbance. An electroencephalogram showed no epileptic paroxysms with normal baseline activity. A cranioencephalic CT scan was repeated 10 days after admission, remaining without lesions. A coronary computed tomography angiography (CCTA) was performed, demonstrating three simultaneous myocardial bridges over the left anterior descending coronary artery (LAD), the right posterior descending coronary artery (RPD), and the right acute marginal coronary artery (RAM). The LAD showed an extensive intramyocardial segment starting at its proximal segment and running adjacent to the right ventricular free wall in the mid-distal portion (not running its usual path through the anterior interventricular sulcus), normalizing its usual anatomical position at the apex level (Figure 1).

**Figure 2:**
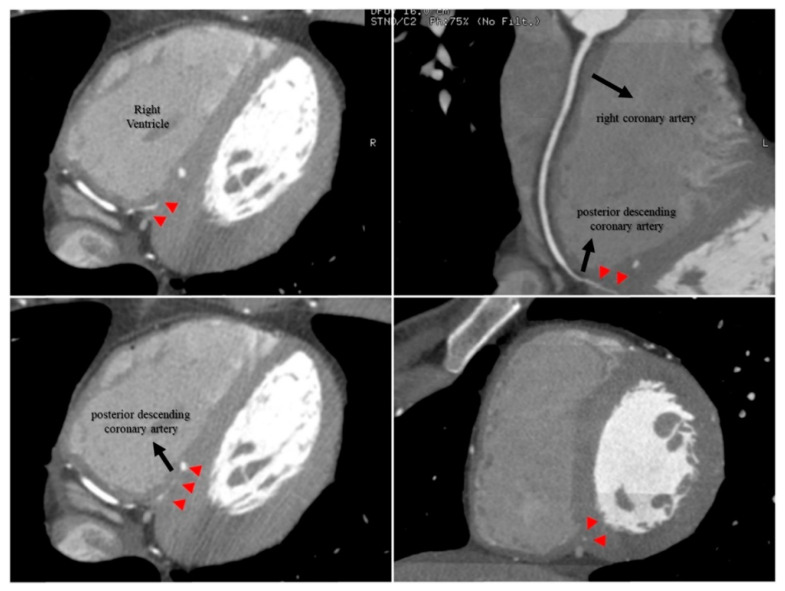
RPD intramyocardial segment shown in CCTA imaging. CCTA showed a short RPD intramyocardial course (red arrowheads) through the posterior interventricular septum instead of traveling its usual course through the posterior interventricular sulcus. CCTA: coronary computed tomography angiography; RPD: right posterior descending coronary artery.

The RPD also did not travel its usual course through the posterior interventricular sulcus, but rather a short intramyocardial course through the posterior interventricular septum (Figure 2).

**Figure 3:**
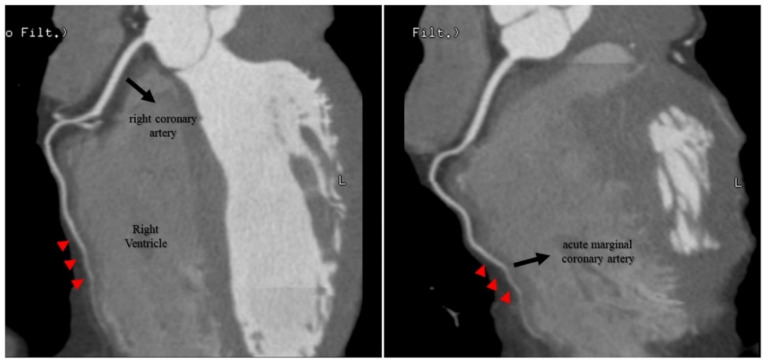
RAM intramyocardial segment shown in CCTA imaging. CCTA demonstrated that the RAM was partially surrounded by myocardium (red arrowheads), being adjacent to the right ventricular cavity in a short extension of its course along the right ventricular free wall. CCTA: coronary computed tomography angiography; RAM: right acute marginal coronary artery.

The RAM was partially surrounded by myocardium, being adjacent to the right ventricular cavity in a short extension of its course along the right ventricular free wall (Figure 3).

**Figure 4:**
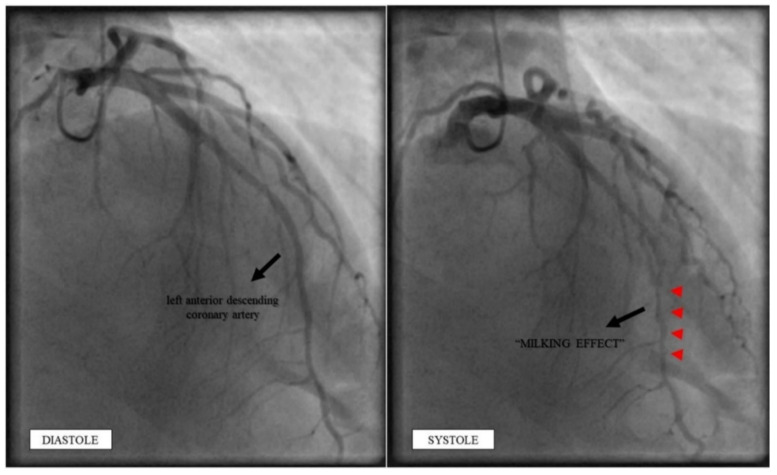
Invasive coronary angiography showing the “milking effect” of myocardial bridging in the mid portion of the LAD. Invasive coronary angiography was performed showing the classic “milking effect” (red arrowheads) of myocardial bridging in the mid portion of the LAD. LAD: left anterior descending coronary artery.

Invasive coronary angiography showed the classic “milking effect” of MB (≥70% collapse during ventricular systole; shown in Figure 4) in the mid portion of the LAD; the left circumflex and right coronary arteries showed no abnormalities.

Cardiac MRI ruled out the presence of cardiomyopathy and myocarditis, and no late gadolinium enhancement was observed. Exercise echocardiography using the Bruce protocol ruled out ischemia: the patient stopped exercise at 15:01 min (15.0 METs) with a maximum heart rate of 173/min, showing no dysrhythmias or conduction disturbances. Tilt testing was negative. An electrophysiological study was performed, detecting neither abnormalities nor induction of arrhythmias.

**Figure 5:**
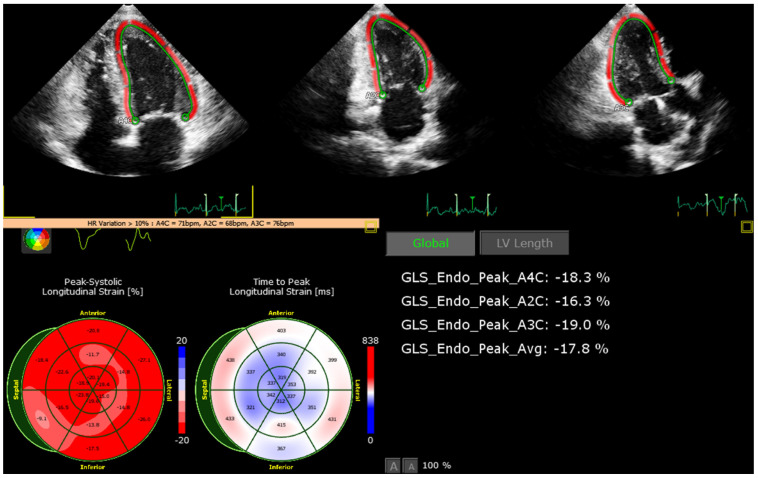
Strain imaging showing an average GLS at the lower limit of normal. Strain imaging was performed revealing that this patient had an average global longitudinal myocardial strain (GLS) at the lower limit of normal (−17.8%). This finding highlights that strain imaging may be a useful tool to investigate myocardial bridging, as it showed diagnostic information otherwise not available by conventional echocardiography. GLS: global longitudinal strain.

Strain imaging was also performed, revealing that this patient had an average global longitudinal myocardial strain (GLS) at the lower limit of normal (GLS −17.8%; shown in Figure 5). This finding highlights that strain imaging may be a useful tool to investigate myocardial bridging, as it showed diagnostic information otherwise not available by conventional echocardiography [6].

### 2.5. Management

Given the absence of ischemia and the patient’s low heart rate, beta-blocker therapy was not initiated. The patient was also not a candidate for percutaneous coronary intervention since he had neither chest pain nor inducible ischemia. Furthermore, after appropriate multidisciplinary Heart Team discussion and patient-shared decision-making, it was decided in favor of a more conservative strategy rather than performing surgery because of: 1—the very high rates of arterial graft failure in CABG surgery (likely due to competitive flow); 2—the fact that myotomy (debridging) is not an innocuous procedure (potential complications include ventricular wall perforation, artery perforation, ventricular aneurysm formation, and postoperative bleeding); 3—the high incidence of late recurrent chest pain after successful debridging in adult patients (up to 60% at 3-year follow-up) [1,7]. The exceptional circumstance of the event (extreme exercise) was also considered in the therapeutic decision. Thus, in the absence of documentation and induction of malignant arrhythmias, it was decided not to place an implantable cardioverter-defibrillator at that time. Conversely, an implantable loop recorder (ILR) was placed for remote monitoring and close follow-up. During hospitalization, the patient remained asymptomatic and was discharged after 18 days. After multidisciplinary discussion and patient-shared decision-making, he refrained from competitive sports and marathon running.

### 2.6. Follow-Up

At 6-months follow-up, the patient remained without complaints. ILR analysis revealed 18 symptomatic episodes that corresponded to sinus tachycardia (120–150/min) without other events. Beta-blocker therapy was initiated. The patient did not return to marathon running.

## 3. Discussion

Exertional syncope is a high-risk complaint in the marathon runner [8]. For most of these runners, the etiology of collapse is exhaustion from the race itself, dehydration, hyponatremia, significant shunting of blood to skeletal muscle, or heat-related illness [8]. Although the prognosis of myocardial bridging is benign, sports medicine recognizes MB as a cause of sudden death among young athletes [2,3,4,5]. The mechanism of ischemia is related to delayed relaxation in early diastole, particularly during physical activity [3,4]. Intense physical activity (such as marathon running) increases the sympathetic tone leading to tachycardia and increased myocardial contractility, thus facilitating ischemia due to MB [3]. In the presence of a long myocardial bridge and prolonged physical activity, myocardial ischemia and life-threatening ventricular arrhythmias can occur [9]. However, a systematic literature review has identified only 35 case reports of cardiac events (including sudden death, myocardial infarction, and arrhythmia) in individuals with MB and in association with physical exertion, suggesting that exercise-related cardiac events associated with MB are extremely rare [2]. So far, only one case has been documented during marathon running, but in a patient with MB and concomitant thrombus in the LAD [2]. Distinctively, the present case refers to a patient admitted for exercise-induced syncope, in whom three simultaneous myocardial bridges were found. Furthermore, strain imaging revealed an average GLS at the lower limit of normal (−17.8%), highlighting the possible usefulness of this echocardiographic tool in this setting. In this illustrative case, we can speculate that the patient’s syncope might probably be associated with an ischemia-induced arrhythmia secondary to MB and potentiated by dehydration in the context of prolonged stress (marathon running).

## 4. Conclusions

Although the prognosis of myocardial bridging is usually benign, this clinical case highlights that MB may be associated with dangerous complications (myocardial ischemia and life-threatening ventricular arrhythmias), particularly during intense physical activity and in the presence of a long myocardial bridge.

## Data Availability

The data that support the findings of this study are available from the corresponding author upon reasonable request.
